# Effect of spinal cord injury upon prostate: adenocarcinoma of prostate in a spinal cord injury patient - a case report

**DOI:** 10.1186/1757-1626-2-9374

**Published:** 2009-12-22

**Authors:** Subramanian Vaidyanathan, Bakul M Soni, Paul Mansour, Peter L Hughes, Gurpreet Singh, Tun Oo

**Affiliations:** 1 Spinal Injuries Unit, District General Hospital, Town Lane, Southport, PR8 6PN, UK; 2 Department of Cellular Pathology, District General Hospital, Southport, PR8 6PN, UK; 3 Department of Radiology, District General Hospital, Southport, PR8 6PN, UK; 4 Department of Urology, District General Hospital, Southport, PR8 6PN, UK

## Abstract

**Introduction:**

Following spinal cord injury, prostate undergoes atrophy probably due to interruption of neuro-hormonal pathways. The incidence of carcinoma of prostate is lower in patients with spinal cord injury above T-10 than in those with lesion below T-10.

**Case presentation:**

A Caucasian male sustained T-4 paraplegia in 1991 at the age of 59-years. He had long-term indwelling urethral catheter. In May 1995, routine blood test showed prostate-specific antigen to be 17.7 mg/ml. Prostate biopsy revealed moderately differentiated primary adenocarcinoma of prostate; Gleason score was 3+3. Bone scans showed no evidence of metastatic bone disease. Bilateral orchidectomy was performed in September 1995. MRI of pelvis revealed no evidence of spread beyond prostatic capsule. There was no pelvic lymphadenopathy. In October 1996, this patient got chest infection and recovered fully after taking amoxicillin. In February 2001, he developed pneumonia and was prescribed cefuroxime intravenously. In March 2001, cystoscopy and electrohydraulic lithotripsy of vesical calculi were carried out. In August 2001, this patient was admitted to spinal unit for management of pressure sores. He expired on 28 June 2002 in local hospital. Cause of death was recorded as acute ventricular failure, congestive heart failure, chronic respiratory failure and spinal cord injury.

**Conclusion:**

Although prostate gland undergoes atrophy in men who sustained spinal cord injury in early age, physicians should be vigilant and look for prostatic diseases particularly in men, who have sustained spinal cord injury during later period of life. Patients with cervical and upper dorsal lesions are at risk of developing potentially life-threatening chest complications after major surgical procedures including radical prostatectomy. Therefore, it may be advisable to consider chemoprevention of prostate cancer with Finasteride, especially in men, who sustained cervical and upper dorsal spinal cord injury during later part of their life.

## Introduction

Frisbie and associates from Spinal Cord Injury Service, Department of Veterans Affairs Medical Centre, West Roxbury, Massachusetts, USA, performed transrectal ultrasonic examination of prostate in spinal cord injury patients. These patients were stratified by severity of paralysis. Ultrasound scan revealed that the prostate gland of severely paralysed spinal cord injury patients was small [1]. Hvarness and associates from Department of Urology, Rigshospitalet at the Copenhagen University in Denmark found that men with spinal cord injury had a smaller prostate than those men without spinal cord injury [2]. Atrophy of prostate following spinal cord injury is probably due to interruption of neuro-hormonal pathways due to extensive cord damage.

Bartoletti and associates from University of Florence, Florence, Italy studied 113 spinal cord injury patients (mean age 61.3) and 109 age-matched able-bodied subjects (mean age 65.4); patients with spinal cord injury were stratified according to age at onset of spinal cord injury. Prostate-specific antigen value and prostate size were significantly lower in patients with spinal cord injury than those observed in able bodied subjects, and an inverse relationship was observed in spinal cord injury patients between these two parameters and patient age at the time of spinal cord injury. No spinal cord injury patient presented with prostate cancer, while 9.7% of control subjects were affected by prostate cancer [3]. Frisbie observed that the incidence of carcinoma of prostate was lower in myelopathy patients with higher levels of paralysis (T10 or above) than in those with lesions at T11 or below [4,5].

We report a Caucasian male with T-4 paraplegia in whom high level of prostate-specific antigen was discovered during routine blood test. Rectal examination revealed enlarged prostate, which was not clinically malignant. But histology of prostate biopsy showed adenocarcinoma. This case illustrates the need to be vigilant not to miss carcinoma of prostate in spinal cord injury patients.

## Case presentation

A 59-year-old, British Caucasian male was working in security and was coming off duty at 0100 hours on 01 March 1991. Five lads tried to ambush the patient and deliberately caused a head-on collision. This patient was wearing a seat belt. He sustained fracture of mandible, left 3 - 8 ribs, left scapula, left humerus, left radius and ulna, right 3 - 6 ribs, right femur, right distal fibula. He developed left haemothorax; chest tube drained 1300 ml of blood. He was ventilated with endotracheal tube. Internal fixation was performed of left humerus, left radius and ulna. External fixation was applied to right femoral shaft. After recovery, he was found to have paraplegia with sensory level at T-4. X-rays of spine and myelogram revealed no abnormality. It was presumed that paraplegia was due to hypotension. Tracheostomy was performed on 19 March 1991. He was weaned off ventilator on 22 April 1991. He developed pressure sore in sacrum. He was managing neuropathic bladder by indwelling urethral catheter.

**Figure 1:**
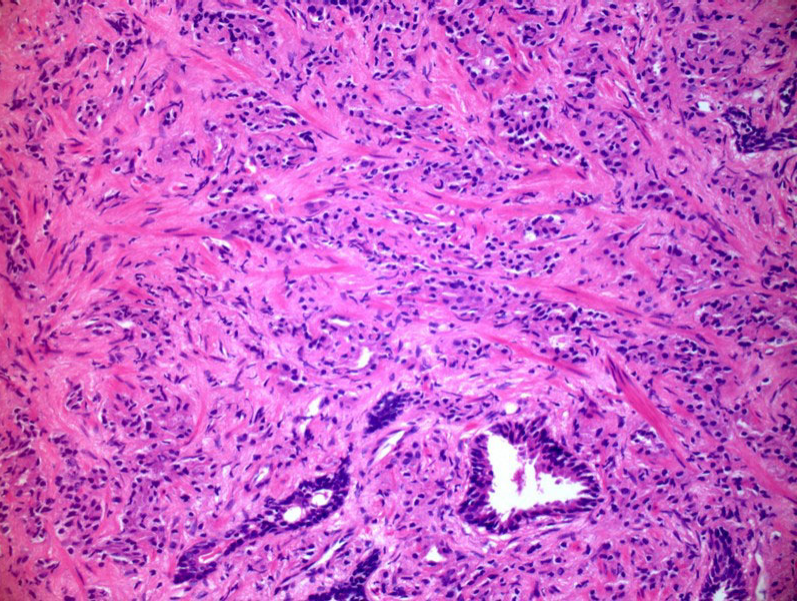
**Core biopsy of prostate (Haematoxylin and Eosin stain) is extensively infiltrated by adenocarcinoma**. The majority of the tumour is composed of poorly formed glands (Gleason pattern 4), contrasting with occasional larger, well-formed, benign glands at bottom.

**Figure 2:**
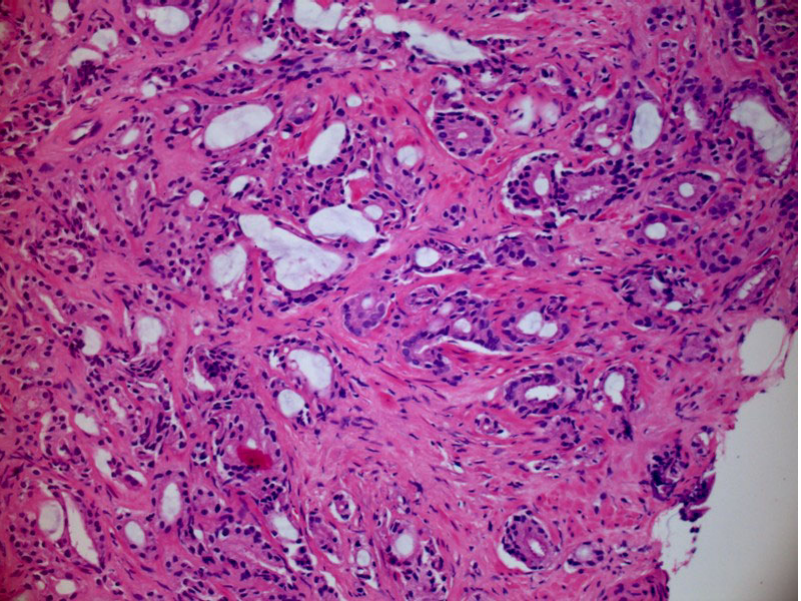
**Core biopsy of prostate (Haematoxylin and Eosin stain) shows small areas of the tumour consist of discrete, relatively well-formed glands with open lumina (Gleason pattern 3)**.

**Figure 3:**
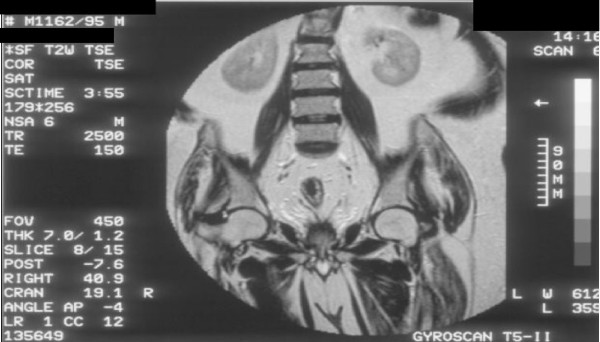
**Magnetic Resonance Imaging of pelvis performed on 23 October 1995: Coronal T-2 weighted image shows no extra-capsular spread of carcinoma of prostate**. Seminal vesicles appear normal.

**Figure 4:**
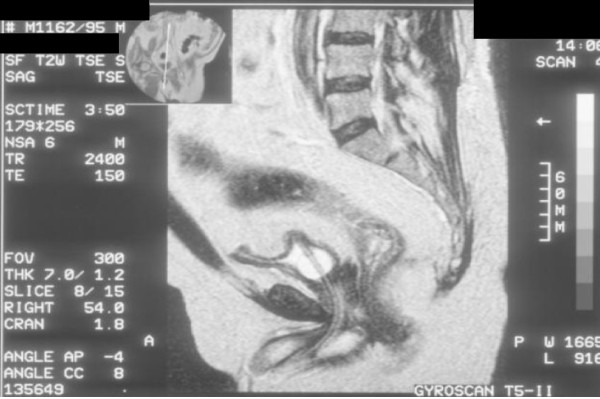
**Magnetic Resonance Imaging of pelvis performed on 23 October 1995: Sagittal T-2 weighted image shows urinary catheter in urethra**. There is no extra-capsular spread of carcinoma of prostate.

**Figure 5:**
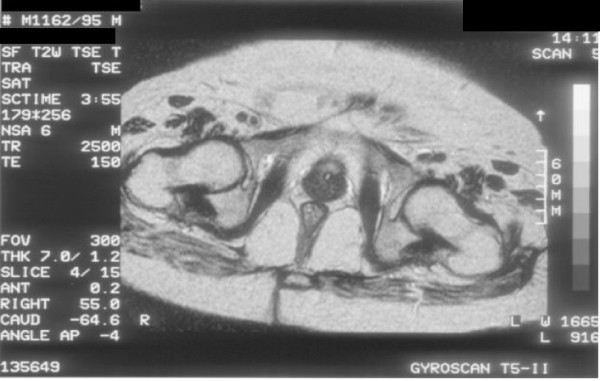
**Magnetic Resonance Imaging of pelvis performed on 23 October 1995: Axial T-2 weighted image shows no extra-capsular spread of carcinoma of prostate**.

On 10 May 1995, during a routine visit to spinal unit, blood sample was sent for Prostate-specific antigen (PSA). Prostate-specific antigen was 17.7 mg/ml. Examination under anaesthesia revealed large, firm prostate but not clinically malignant. Biopsies were taken on 12 September 1995. All cores of prostatic tissue were infiltrated by a moderately differentiated primary adenocarcinoma of prostate Gleason score 3+3. (Figures 1 and 2) Focal perineural invasion was present. Bone scan was done on 18 September 1995, which showed no evidence of metastatic bone disease. Bilateral orchidectomy was performed on 19 September 1995. Microscopy revealed normal testes. MRI of pelvis, carried out on 23 October 1995, revealed no evidence of any spread of prostatic malignancy beyond the prostatic capsule. (Figures 3, 4 and 5) The prostate measured 3 cm in diameter and appeared well defined from rectum, levator ani muscles and seminal vesicle. There was no pelvic lymphadenopathy.

In February 1996, this patient sustained injury to right ankle while he was hoisted. X-ray showed fracture of distal tibia and fibula. In October 1996, he developed chest infection and was prescribed amoxicillin. In February 2001, this patient developed pneumonia. He received cefuroxime intravenously. He was experiencing repeated catheter blocks. Flexible cystoscopy showed several calculi in urinary bladder. In March 2001, cystoscopy and electrohydraulic lithotripsy of vesical calculi were carried out. Prostate-specific antigen levels during this patient's follow-up are given in Table 1. In August 2001, this patient was admitted to spinal unit for management of pressure sores. He expired on 28 June 2002 in local hospital. Cause of death was recorded as acute ventricular failure, congestive heart failure, chronic respiratory failure and spinal cord injury.

**Table 1:** Prostate-specific antigen levels during follow-up after this patient underwent bilateral orchidectomy on 19 September 1995.

Date	Prostate-specific antigen (ng/mL)
10 May 1995	17.7
11 September 1995	21.8
23 October 1995	1.9
16 January 1996	2.5
07 February 1996	2.7
25 March 1996	1.8
19 June 1996	1.9
11 September 1996	2.2
23 October 1996	2.3
15 January 1997	1.7
15 April 1997	1.6
15 July 1997	1.3
21 October 1997	1.0
20 January 1998	1.4
20 April 1998	1.7
06 February 2001	2.4
07 September 2001	2.9

## Discussion

In an elderly veteran population, digital rectal examination was found to result in a statistically significant but clinically insignificant increase in serum PSA level [6]. Ornstein and associates from Division of Urologic Surgery, Washington University School of Medicine, St. Louis Missouri, USA observed that biological variation for total and free prostate-specific antigen was 14.7 and 14.0%, respectively. At 1 hour after rectal examination total and free prostate-specific antigen increased by more than the biological variation in 31 and 48 per cent of the men, respectively [7]. Increases were significantly greater in men whose initial prostate-specific antigen concentrations were less than 4.0 ng/mL. There was a dramatic increase in total and percentage of free prostate-specific antigen in all men 1 hour after prostatic biopsy. Total prostate-specific antigen remained elevated for at least 1 week in most men, while percentage of free prostate-specific antigen returned to within or less than the biological variation of the baseline level in 90% of the men by 24 hours.

Some spinal cord injury patients undergo manual evacuation of bowels or digital check of rectum to avoid bowel accidents; these procedures can lead to transient elevation of prostate-specific antigen level in blood. Therefore, a time gap of 48 hours should be allowed before prostate-specific antigen is checked in these patients. Otherwise, men with tetraplegia and paraplegia, in whom manual evacuation of bowels is performed, or digital check of rectum is carried out twice or three times a day may be subjected to unnecessary prostate biopsies just because prostate-specific antigen level was above the upper limit of laboratory reference range.

Patients with cervical or upper dorsal spinal cord injury have limited cardio-respiratory reserve. Therefore, these patients are at high risk for developing serious pulmonary complications after major surgery such as radical prostatectomy. Our patient was not considered for radical prostatectomy for the same reason. Tetraplegic subjects and those with upper dorsal lesions are at risk of developing potentially life-threatening complications after major surgical procedures including radical prostatectomy. Therefore, it may be advisable to consider chemoprevention for prostate cancer, especially in men, who sustained cervical and upper dorsal spinal cord injury during later part of their life. Recently, the Prostate Cancer Prevention Trial enrolled 18 882 men aged > or = 55 years with a prostate-specific antigen level of < 3.0 ng/mL and normal digital rectal examination findings, and randomized them to Finasteride 5 mg daily or placebo [8]. Prostate cancer data from evaluable biopsies obtained within 7 years plus < or = 90 days of randomization were examined. Finasteride significantly reduced the risk of prostate cancer relative to placebo across multiple Gleason scores (4 through 7), including a 58% reduction in Gleason score 5 prostate cancer risk (P < .0001), a 52% reduction in Gleason score 6 prostate cancer risk (P < .0001), and a 22% reduction in Gleason score 7 prostate cancer risk (P = .0368). Re-evaluation of the results based on the pathology of the radical prostatectomy specimens and longer follow-up showed a 30% reduction in cancer incidence with Finasteride and no significant differences in Gleason scores compared with placebo [9]. Modelling studies suggest that with Finasteride, the risk of high-grade cancer is unchanged or reduced. Sexual dysfunction and gynecomastia were observed but the rates were low [10].

## Conclusion

Although prostate gland undergoes atrophy in men who sustained spinal cord injury in early age, physicians should be vigilant not to miss carcinoma of prostate particularly in men, who have sustained spinal cord injury during later period of life. Some spinal cord injury patients undergo manual evacuation of bowels or digital check of rectum to avoid bowel accidents; these procedures can lead to transient increase in prostate-specific antigen level in blood. This should be borne in mind otherwise, some of these patients, who undergo manual evacuation of bowels, or digital check of rectum twice or three times a day may be subjected to unnecessary prostate biopsies just because prostate-specific antigen level is above the upper limit of laboratory reference range. Tetraplegic subjects and those with upper dorsal lesions are at risk of developing potentially life-threatening complications after major surgical procedures including radical prostatectomy. Therefore, it may be advisable to consider chemoprevention of prostate cancer with Finasteride, especially in men, who sustained cervical and upper dorsal spinal cord injury in later part of their life.

## Consent

Unfortunately, this patient had left us for heavenly abode several years ago. The spinal unit lost touch with the patient's family as well. Therefore, it is not possible to obtain written informed consent from the patient or patient's next of kin for publication of this case report and accompanying images. We do not think that neither the patient nor his relatives would have objected to presentation of this case in Cases Journal, as primary purpose of publication is education of health professionals leading to improved quality of care of spinal cord injury patients.

## Competing interests

The authors declare that they have no competing interests.

## Authors' contributions

SV carried out routine review of this patient, took blood sample, which revealed raised PSA level; discussed the diagnosis of cancer of prostate with the patient and his relatives; performed bilateral orcidectomy; developed the concept of this article and wrote draft; BMS was the Consultant in charge of this patient; PM reported prostate biopsy and orchidectomy specimen; PH reviewed MRI of pelvis. All authors read and approved the final manuscript.
